# Minimal important change (MIC): a conceptual clarification and systematic review of MIC estimates of PROMIS measures

**DOI:** 10.1007/s11136-021-02925-y

**Published:** 2021-07-10

**Authors:** Caroline B. Terwee, John Devin Peipert, Robert Chapman, Jin-Shei Lai, Berend Terluin, David Cella, Pip Griffiths, Lidwine B. Mokkink

**Affiliations:** 1 Department of Epidemiology and Data Science, Amsterdam Public Health Research Institute, Amsterdam UMC, Vrije Universiteit Amsterdam, P.O. box 7057, 1007 Amsterdam, MB The Netherlands; 2 Department of Medical Social Sciences, Northwestern University Feinberg School of Medicine, Chicago, IL USA; 3 Department of General Practice, Amsterdam Public Health Research Institute, Amsterdam UMC, Amsterdam, The Netherlands; 4 Patient Centered Endpoints, IQVIA, Reading, UK

**Keywords:** Minimal important change, Interpretation, Patient-reported outcomes, PROMIS, Methodology

## Abstract

We define the minimal important change (MIC) as a threshold for a *minimal* within-person *change* over time above which patients perceive themselves *importantly* changed. There is a lot of confusion about the concept of MIC, particularly about the concepts of minimal *important* change and minimal *detectable* change, which questions the validity of published MIC values. The aims of this study were: (1) to clarify the concept of MIC and how to use it; (2) to provide practical guidance for estimating methodologically sound MIC values; and (3) to improve the applicability of PROMIS by summarizing the available evidence on plausible PROMIS MIC values. We discuss the concept of MIC and how to use it and provide practical guidance for estimating MIC values. In addition, we performed a systematic review in PubMed on MIC values of any PROMIS measure from studies using recommended approaches. A total of 50 studies estimated the MIC of a PROMIS measure, of which 19 studies used less appropriate methods. MIC values of the remaining 31 studies ranged from 0.1 to 12.7 T-score points. We recommend to use the predictive modeling method, possibly supplemented with the vignette-based method, in future MIC studies. We consider a MIC value of 2–6 T-score points for PROMIS measures reasonable to assume at this point. For surgical interventions a higher MIC value might be appropriate. We recommend more high-quality studies estimating MIC values for PROMIS.

**Supplementary Information:**

The online version contains supplementary material available at 10.1007/s11136-021-02925-y.

## Introduction

There are several ways to interpret change scores arising from patient-reported outcome measures (PROMs). One possible threshold is the minimal important change (MIC) estimate, which refers to the smallest change in score that patients consider important. The MIC is the lower bound of a distribution of thresholds for important change. There is a lot of confusion about the concept of MIC, which questions the validity of published MIC values [1, 2]. First, there is inconsistency in terminology used (e.g., minimal important change, minimal important difference, minimal clinically important difference, meaningful change threshold, to name a few). Similar terms may refer to different concepts and vice versa. Second, there is particular confusion about the concepts of minimal *important* change and minimal *detectable* change, which refer to different concepts [3, 4]. Third, there are differences in methods used for estimating the MIC, some more and some less methodologically sound [5]. This confusion hampers and may even bias the interpretation of PROM change scores in research and clinical practice.

An increasingly used, innovative set of PROMs is the Patient-Reported Outcomes Measurement Information System (PROMIS^®^). It covers domains of health-related quality of life (HRQOL), such as pain, fatigue, physical function, anxiety, depression, and the ability to participate in social roles and activities, that are commonly important for adults and children with and without (chronic) medical conditions [6, 7]. Most PROMIS measures are rooted in item response theory (IRT)-based item banks (i.e., large sets of calibrated questions measuring the same domain (construct)), which enables efficient measurement through fixed-length short forms and/or computerized adaptive testing (CAT) [8–10]. A number of studies have estimated MIC values for PROMIS measures. However, in light of its increasing use across the world [11–19], and the aforementioned confusion in the interpretation literature, additional guidance is needed on interpreting PROMIS change scores.

The aims of this study were: (1) to clarify the concept of MIC and how to use it; (2) to provide practical guidance for estimating methodologically sound MIC values; and (3) to improve the applicability of PROMIS by summarizing the available evidence on plausible PROMIS MIC values.

## Part 1: the concept of MIC and how to use it

We define the MIC as a threshold for a minimal within-person change over time above which patients perceive themselves importantly changed. Assuming that all patients have their individual threshold of what they consider a minimal important change, the MIC can be conceptualized as the mean of these individual thresholds [20, 21]. This definition of MIC is made up of three important elements: first, it refers to a threshold for a *minimal* change above which patients perceive themselves as changed (improved or deteriorated). Second, it refers to a change that is considered *important* to patients. And third, it refers to a within-patient *change* over time.

These three elements do not only define what the MIC is but also clarifies what the MIC is not. The MIC does not refer to thresholds for changes that are considered more than minimal (e.g., a mean change in patients who reported to be “much better” is not a MIC). There are other relevant concepts that reflect meaningful change thresholds that are larger than minimal, such as Clinically Significant Change [22], Sufficiently Important Difference [23] or Smallest Worthwhile Effect [24]. These concepts are outside the scope of this paper.

Next, the MIC is not a minimal *detectable* change (MDC, also referred to as smallest detectable change (SDC)). The MDC is the smallest change in score than can be detected statistically with some degree of certainty (e.g., 95 or 90%), based on the standard error of measurement (SEM) or limits of agreement from a test–retest reliability design. The MDC does not relate to the importance of change to the patients under investigation [4, 25–27]. The MDC is also an important benchmark for interpreting PROM change scores, but it is also outside the scope of this paper.

Finally, the MIC is not a *difference* between (groups of) patients. For example, a difference between patients who reported to be “a little better” and those who reported to be “about the same” refers to a minimal important *difference* (MID), not a minimal important within-person *change* (MIC). The MID is another relevant benchmark for interpreting PROM scores but is also outside the scope of this paper.

The MIC, as defined above, can be used for different purposes. In research, some use the MIC value as a threshold to determine the number of *responders* in clinical trials or other studies (i.e., patients who have a change at least as large as the MIC value) [28, 29]. This responder definition adds a meaningful interpretation to study results from the patients’ perspective. In clinical practice, the MIC value can also be used to determine the number of responders in groups of patients who receive certain treatments to inform future patients about the expected effects of treatments. For example, a patient can be told that about 70% of patients experience a minimal important change after a given treatment. This may facilitate shared-decision making. However, it is necessary to acknowledge that the estimated MIC value is derived from a wider sample of patients, and the threshold may not apply to the individual patient in the clinical trial or in the consultation room. If a responder is defined as an individual whose PROM change score exceeds the MIC, then on a group level the percentage of responders will probably be correct. However, this doesn’t mean that all patients have been classified correctly, based on their individual PROM change score being smaller or greater than their individual MIC. This is because all patients have their own individual threshold of what they consider a minimal important change [20]. Furthermore, measurement error in the PROM change score further contributes to misclassification of individuals.

In addition to being used as a threshold for responder definitions, the MIC value can be used as a probabilistic value, rather than a deterministic cut-point, by clinicians to interpret change scores in light of the probability that an individual patient has experienced a meaningful change. For example, if the estimated MIC value of a PROM is 10 points and an individual patient has changed more than 10 points, it is more likely that the patient has importantly improved than that the patient has not importantly improved. This might help the clinician start a conversation with the patient.

## Part 2: guidance for estimating MIC values

A variety of methods have been used in the literature to estimate MIC values [1, 30, 31]. Many methods, however, do not refer to the concept of MIC as described above. MIC methods are often categorized into distribution-based and anchor-based methods. Distribution-based methods use statistical parameters, such as a standard deviation (SD) or standard error of measurement (SEM) for estimating the MIC value. These parameters refer to measurement error (minimal *detectable* change) but do not relate to the *importance* of the change to the patients under investigation and, while they add useful context to interpreting MIC values, they do not capture the spirit of the MIC [3, 4, 27].

Anchor-based methods are generally more appropriate because they relate change scores on the instrument of interest to an external criterion of *important* change. Often, a single question at follow-up is being used as the external criterion (the anchor), asking patients how much they have changed, for example on a global 5- or 7-point rating scale ranging from “much worse” to “much better”. The most simple and prevalent method used to estimate the MIC value is the *mean change method,* where the MIC value (further referred to as MIC_mean_) is defined as the change score on the measure of interest in the subgroup of patients that reported to be “a little better” (minimal important improvement) or “a little worse” (minimal important deterioration) on the anchor question [32]. Studies have shown that a MIC for improvement may not be the same as a MIC for deterioration [33–35]. The mean change method has some important drawbacks. First, the subgroup of patients who reported to be “a little better” is often small, which results in imprecise MIC_mean_ estimates. More importantly, the MIC_mean_ value does not reflect a threshold for minimal improvement because it is defined as the *mean* of the entire group of patients who reported to be “a little better”. As all patients in this group reported to be minimal importantly changed on the anchor, the mean change in score on the PROMs of interest in this group of patients is higher than the threshold for minimal important change. Finally, it has been shown that if the anchor is not completely accurate, MIC_mean_ estimates are more severely biased than other anchor-based methods and will always be biased downwards [36].

Two additional, more appropriate, anchor-based MIC methods are the ROC method and the MIC predictive modeling method, which are described in more detail below and in Online supplement 2. In addition, a relatively new qualitative method, based on comparing vignettes (descriptions of health status of hypothetical patients), is also described below.

### ROC method

The Receiver Operating Characteristic (ROC) curve method is based on the ability of a measure to distinguish patients who reported to be improved from patients who reported to be not improved (i.e., stayed the same or worsened) on the anchor. The MIC value (further referred to as MIC_ROC_) is most often defined as the value for which the sum of the proportions of misclassifications ([1-sensitivity] + [1-specificity]) is smallest [32]. An advantage of this method is that it uses the entire study sample, leading to more reliable estimates than the MIC_mean_. Moreover, it estimates the *threshold* between ‘not changed’ and ‘a little better’ (minimal important improvement) or ‘a little worse’ (minimal important deterioration). A disadvantage is that the MIC_ROC_ will be biased if the percentage of improved patients is not 50% [20].

### Predictive modeling method

The predictive modeling approach is based on the predicted probability that a patient belongs to the improved group (based on the anchor) given the observed change score [21]. This method uses logistic regression analysis with the group variable (improved versus not improved [stayed the same and worsen] on an anchor) as the dependent variable and the change score on the instrument of interest as the independent variable. The MIC value (further referred to as MIC_predict_) is defined as the change score associated with a likelihood ratio of 1, which is the change score where the posttest probability of belonging to the improved group (i.e., after knowing the patient’s PROM change score) equals the pretest probability of belonging to the improved group (before knowing the patient’s PROM change score, the pretest probability is the percentage of improved patients in the sample) [20, 21]. The MIC_predict_ is more precise than the MIC_ROC_ and a formula has been published to correct the MIC_predict_ for bias if the percentage of improved patients is not 50% [20]. It is therefore considered as a better option than the MIC_ROC_. In Online supplement 2 we provide additional details and SPSS and R codes (See also [37]) for how MIC_ROC_ and MIC_predict_ can be calculated.

### Vignette-based method

The anchor-based MIC methods described above depend on the reliability and validity of the anchor question, which has been criticized [30, 38, 39]. An alternative method for instruments with IRT-based scores is a vignette-based method, often referred to as bookmarking or standard setting. With this method, patients are asked to compare vignettes (descriptions of health status of hypothetical patients) in focus groups or in a survey [40–42]. Each vignette represents a health status with an associated score on the underlying IRT metric. Patients are asked to indicate whether a hypothetical change in health status from one vignette to another would be considered an important change. The MIC (further referred to as MIC_vignette_) has been defined as the mean difference in scores between pairs of vignettes that represent a minimal important change. If the mean difference is used to estimate the MIC_vignette_, this method may suffer a similar issue to MIC_mean_ in that it represents a value higher than the minimal threshold. Alternatively, it would also be possible to ask patients to rate the change between two (or more) vignettes on an anchor question and then use the predictive modeling method to estimate the MIC_predict_.

In Box 1 we provide a summary of general recommendations for the design and analysis of MIC studies.

Box 1: Recommendations for conducting and reporting MIC studies

1. The predictive modeling or ROC method should be used over the mean change method because MIC_predict_ and MIC_ROC_ provide a *threshold* between improved and not improved patients [21], while the MIC_mean_ does not reflect a threshold for minimal improvement, but rather a *mean* in a subgroup of patients who considered themselves as minimally improved. The MIC_predict_ is more precise than the MIC_ROC_ and can be corrected for bias if the percentage of improved patients is not 50%, and is therefore recommended as best option. Vignette-based methods can also be considered or used in addition to the MIC_predict_ or MIC_ROC_ because they do not require a longitudinal study. The MIC_vignette_ is typically determined in a qualitative or survey study [40, 97].
2. The MIC_predict_ or MIC_ROC_, should be determined in a longitudinal study, where patients complete the instrument of interest at baseline and again after a relevant time period (e.g., after an intervention). The most efficient design is one in which about half of the patients are expected to change (at least to a minimal important degree) on the domain of interest (e.g., physical function) and about half of the patients are expected not to change. If an intervention is applied between baseline and follow-up measurement, this intervention should be clearly described.
3. An anchor question should be completed by the patients at follow-up. The anchor question should measure the same construct as the instrument of interest. For example, for estimating the MIC of a fatigue instrument, the anchor question should state “how much has your fatigue changed since …”. The anchor question should refer to a change since the previous measurement (e.g., since before treatment). The anchor question can have 3–7 response options, ranging from “much worse” to “much better”. Patients who report to be “a little better” or more will be included in the improved group, while the rest of the patients will be included in the not improved group.
4. The sample size of the MIC study should be at least 100 patients [2]. Ideally, the percentage of patients in the improved group should be 50%. The percentage of improved patients should be reported. If the percentage of improved patients is not about 50%, the adjusted MIC_predict_ should be used (see Online supplement 2) [20].
5. We recommend to plot the distributions of change scores in the improved group and in the not improved group (see Online supplement 2) to visualize how well the instrument of interest can distinguish between the improved and not improved patients [32].
6. The correlation between the change score on the instrument of interest and the anchor question should be at least 0.30 to assume validity of the anchor [2]. This correlation should be reported. If the correlation is too low, the data are not suitable for estimating MIC value.
7. A 95% confidence interval around the MIC value should also be calculated and reported (see Online supplement 2) [98].

## Part 3: evidence on plausible MIC values of PROMIS measures

To summarize the available evidence on plausible MIC values of PROMIS measures we performed a search in PubMed from inception up to May 31, 2021 to identify all studies that estimated the MIC of one or more PROMIS measures.

### Methods

We extracted relevant search terms from the COSMIN PubMed filter for finding studies on measurement properties [43]. The full search strategy is presented in Online supplement 3. One author (CBT) screened the abstracts.

We included studies that determined a MIC value for any PROMIS measure (adults and pediatric, any domain, any language, any version (e.g., v1.0, v2.0), full bank, short form or CAT) in any population. We extracted the following information: PROMIS measure(s) used (including domain, version number, administration type, language, age version) and country in which data were collected, study population, intervention(s), length of follow-up, sample size on which the MIC values(s) was/were based, MIC methods used, correlation between PROMIS change scores and the anchor (Spearman correlation if presented, otherwise Pearson correlation), percentage of patients improved based on the anchor (only for studies estimating MIC_ROC_ or MIC_predict_), and MIC values.

We only extracted MIC values based on anchor-based methods or vignette-based methods. We did not extract distribution-based MIC values. We only extracted MIC values based on longitudinal anchors, referring to within-person change over time. We did not extract values based on cross-sectional anchors, referring to minimal important differences between groups of patients (e.g., difference between patients who reported to be “slightly improved” and patients who reported to be “not changed” [44] or differences between patients with different levels of disease [45]) because these values refer to a minimal important *difference* (MID) rather than a minimal important *change* (MIC). When MIC values of other instruments were used as an anchor, we checked whether these MIC values were based on anchor-based methods. Furthermore, we did not extract MIC values that referred to more than a minimal important change (for example, MIC_mean_ values based on mean changes in patients who reported to be “much better” were not included). We extracted MIC values for minimal important improvement and for minimal important deterioration separately. MIC values determined in groups of less than 10 patients were not extracted. Data extraction was initially performed by one author (either JDP, RC, PG, or CBT) for each paper, and extracted data were checked by another author (CBT or LBM). Missing information (for example, regarding the version numbers of PROMIS measures used) was requested by email (by CBT) to the primary authors of the papers.

All PROMIS measures are scored on a T-score metric, in which 50 is the mean of a relevant reference population (often a general population) with a standard deviation (SD) of 10. Higher scores mean more of the concept being measured (e.g., worse fatigue, better physical function).

### Results

The search yielded 911 abstracts, including 50 studies that estimated a MIC value of a PROMIS measure [41, 44–92]. All studies used self-reported PROMIS data, no studies on proxy-reported data were found. Of these 50 studies, 10 studies used only distribution-based methods [49, 50, 52, 55, 58, 66, 68, 74, 75, 77]; five studies estimated a minimal important *difference* (MID) rather than minimal important *change* (MIC) [44, 62, 63, 72, 73]; one study averaged estimates based on cross-sectional and longitudinal anchors as well as distribution-based estimates [84]; one study estimated a MIC value that referred to more than a minimal important change [92]; and two studies intended to calculate an anchor-based MIC but reported only a distribution-based MIC because the area under the ROC curve was considered too low [82, 83]. Data from these 19 studies were not extracted.

MIC values from the remaining 31 studies were extracted and presented in Tables 1, 2, 3 (See also Tables S1 through S11 in Online supplement 1) [41, 44–48, 51, 53, 54, 56, 57, 59–61, 64, 65, 67, 69–71, 76, 78–81, 85–91]. Twenty-eight of these 31 studies used anchor-based methods. Anchor-based MIC values from these studies were extracted. Distribution-based MIC values that were also presented in 17 of these studies were not extracted [45–48, 53, 54, 59, 64, 69–71, 78, 81, 85–87, 91], three MIC values based on cross-sectional anchors were not extracted [45, 46, 48], and one MIC value based on patients who experienced a “meaningful change” (more than minimal) was also not extracted [53]. Out of the 28 anchor-based studies, 24 used (a variation of) a mean change method [45–48, 51, 53, 54, 55, 59, 60, 64, 65, 69–71, 76, 78, 79, 85–89, 91], five studies used an ROC method [53, 54, 56, 67, 81], of which two studies used both methods [53, 54], and one study used the predictive modeling method [90]. In addition to the 28 studies that used anchor-based methods, the MIC values of three studies that used a vignette method to estimate MIC values were also extracted [41, 61, 80].

**Table 1:** Minimal important change values for adult PROMIS pain interference

References	PROMIS measure	Language	Population	Intervention	Method used	Follow-up	N^a^	Correlation of PROMIS change score with anchor	MIC value^b^	Comments
Amtmann et al. [46]	V1.0 pain interference CAT	English (US)	Low back pain	Epidural steroid injection or antidepressants, psychotherapy, or both	Mean change in patients that reached an MIC on other PROMs (2–3 point positive or negative change on NRS average and worse pain intensity, 5–10 points on RMDQ, 0.5–1.0 × SD on BPI)	1 Month	Subgroups of 414	Not reported	3.5–5.5	Sample size on which MIC is based not reported Anchor for BPI distribution-based Improved and deteriorated patients combined
Bartlett et al. [78]	V1.0 pain interference 8a V1.0 pain interference 4a	English (US)	Rheumatoid arthritis	Medication	Mean change of individuals responding a little better or a little worse on an anchor question on change in pain interference	4.6 (2.4) Months	51	Not reported	Improvement: 1.1	
							60		Deterioration: 1.8	
							50		Improvement: 1.1	
							59		Deterioration: 1.7	
Beaumont et al. [79]	V1.1 pain interference 6b	English (US)	Rheumatoid arthritis	Not reported	Mean change of individuals responding a little better or a little worse on an anchor question on response to treatment	6 Months	60	0.24	Improvement: 1.9	
							126		Deterioration: 0.6	
						12 Months	53	0.29	Improvement: 1.8	
							122		Deterioration: 1.5	
Bernstein et al. [47]	V1.0 pain interference CAT	English (US)	Carpal tunnel release	Surgery	Mean change in subgroup that reached an MIC on MHQ Pain (13 points) or BCTQ (0.74 points)	6 Weeks or 3 months	52 (MHQ)	Not reported	8.9	MIC of MHQ pain was based on MHQ satisfaction, where ‘satisfied’ was defined based on effect size (distribution-based)
							40 (BCTQ)		9.7	
Bingham et al. [80]	V1.1 pain interference	English (US)	Rheumatoid arthritis	NA	Qualitative bookmarking method	NA	11	NA	Improvement: 10	
									Deterioration: 10	
Chen et al. [48]	V1.0 pain interference 4a, 6a, 6b, 8a	English (US)	Chronic low back pain	Pharmacological or behavioral approaches	Mean change scores corresponding to a 1-point change in prospective change in global rating of pain (range − 1 to +4)	6 Months	Subgroups of 261	0.23–0.49	Improvement: 2.3	Sample size on which MIC is based not reported
									Deterioration: 3.8	
					Mean change scores corresponding to one category shift on an anchor question on change in pain;				Improvement: 2.4	
									Deterioration: 1.9	
Chen et al. [48]	V1.0 pain interference 4a, 6a, 6b, 8a	English (US)	Chronic back pain or hip or knee osteoarthritis pain	Opioid therapy or non-opioid medication	Mean change scores corresponding to a 1-point change in prospective change in global rating of pain (range − 1 to +4)	3 Months	Subgroups of 240	0.23–0.49	Improvement: 3.8	Sample size on which MIC is based not reported
									Deterioration: 3.8	
					Mean change scores corresponding to one category shift on an anchor question on change in pain				Improvement: 2.4	
									Deterioration: 2.4	
Chen et al. [48]	V1.0 pain interference 4a, 6a, 6b, 8a	English (US)	Stroke survivors	Stroke self-management	Mean change scores corresponding to a 1-point change in prospective change in global rating of pain (range − 1 to +4)	3 Months	Subgroups of 258	0.23–0.49	Improvement: 3.1	Sample size on which MIC is based not reported
									Deterioration: 5.6	
					Mean change scores corresponding to one category shift on an anchor question on change in pain				Improvement: 2.0	
									Deterioration: 3.1	
Forlenza et al. [81]	Pain interference CAT	English (US)	Biceps tenodesis	Surgery	Optimal ROC cut-off point to distinguish patients who reported improvement from patients who reported no improvement on an anchor question on overall function of the shoulder	7.6 (6.0–9.3) months	112	Not reported	5.6	
Hung et al. [53]	V1.1 pain interference CAT	English (US)	Foot and ankle disorders	Surgery	Optimal ROC cut-off point to distinguish patients who reported much worse, worse, improved and much improved from patients who reported slightly worse, no change, slightly improved	3 Months	64 vs 41 (61% changed)	Not reported	8.0	
						> 3 Months	321 vs 187 (63% changed)		12.4	
						> 6 Months	170 vs 100 (63% changed)		5.5	
Katz et al. [69]	V1.1 pain interference 4a	English (US)	Rheumatic diseases (> 93% RA)	Not reported	Mean change of individuals responding somewhat better or somewhat worse on an anchor question on change in pain interference. MIC also calculated per baseline category of NRS pain (low, moderate, high)	Average of three periods of 6 months	± 400	Not reported	Improvement: Total: 1.7 Low baseline pain: 0.4 Moderate baseline pain: 2.2 High baseline pain: 4.1	Each patient was included three times in the analyses
							> 550		Deterioration: Total: 1.8 Low baseline pain: 5.0 Moderate baseline pain: 1.7 High baseline pain: 0.4	
Katz et al. [70]	V1.1 pain interference 4a	English (US)	SLE	Not reported	Mean change of individuals responding somewhat better or somewhat worse on an anchor question on change in function	6 Months	29	Not reported	Improvement: 2.0	
							37		Deterioration: 1.9	
Kenney et al. [56]	Pain interference CAT	English (US)	Patients undergoing knee arthroscopy	knee arthroscopy	Optimal ROC cut-off point to distinguish patients who reached a MIC (11.5 points on 0–100 scale) on the IKDC	2 Weeks to 12 months	51 vs 25 (67% improved)	− 0.67	3.2	MIC (slightly) overestimated because percentage of improved patients > 50%
Khutok et al. [87]	Pain interference 4a (part of PROMIS-29 v2.1)	Thai	Chronic low back pain	Many received standard physical therapy	Mean change of individuals reporting little improvement on an anchor question on change in pain interference	4 Weeks	49	0.19	1.6	
Kuhns et al. [88]	Pain interference CAT	English (US)	Patients undergoing hip arthroscopy, (including 61 athletes)	Hip arthroscopy	Optimal ROC cut-off point to distinguish patients who reported their level of function after surgery excellent or good versus patients who reported their level of function after surgery average, fair or poor	21.3 (± 4.4) months	113	Not reported	10.9	Anchor question about current (postoperative) level of functioning instead of change
Lee et al. [60]	V1.0 pain interference 6b	English (US)	Adults (40 +) with Knee OA	Tai Chi or physical therapy	Mean change in patients that reached 1–2 MICs on SF-36 subscale	12 Weeks	20–30	0.53	2.4	Unclear which MIC values for SF-36 were used and whether they were anchor-based Lower bound of MICs was increased to > SEM
Schwartz et al. [65]	V1.0 pain interference 6b	English (US/Canada)	Disc herniation or spinal stenosis	Surgery	Mean change at each follow-up time point for patients who reported being somewhat better	Baseline-6 weeks	32	Not reported	6.5	
						6 Weeks-3 months	32		0.7	
						3–6 Months	23		1.8	
						6–12 Months	16		0.9	
Stephan et al. [99]	V1.0 pain interference 4a	German (Switzerland)	Foot and ankle disorders	Orthopedic foot and ankle surgery	Optimal ROC cut-off point to distinguish patients who reported operation did help or operation helped a lot from patients who reported operation helped only a little, did not help or made things worse	6 Months	166 vs 36 (82% improved)	0.45	5.0	Anchor does not refer to change in pain interference MIC overestimated due to high percentage of patients improved
Yost et al. [45]	V1.1 Cancer Pain Interference− 10	English (US)	Advanced-stage cancer	Chemotherapy only (74.3%) Chemo- and radiation therapy (9.9%) Other mixed modalities (13.8%) Missing 2.0%	Mean change in patients who changed 2–3 points on an anchor with range – 10 to 10 Mean change in patients who changed 1–2 MICs on BPI [0.5 × SD (1.4 points)] Mean change in patients who reported a little better or moderately better or a little worse or moderately worse	6–12 Weeks	Subgroups of 88	> 0.30	Median 3.5 (range 2.2–4.8)	PROMIS Cancer scales are on the same metric as the PROMIS generic item banks Estimates for improvement and deterioration were lumped together MIC on BPI distribution-based

*BCTQ* boston carpal tunnel questionnaire, *BPI* brief pain inventory, *CAT* computerized adaptive testing, *IKDC* international knee documentation committee; *MHQ* Michigan hand questionnaire, *MIC* minimal important change, *NA* not applicable, *NRS* numerical rating scale, *OA* osteoarthritis, *PROMs* patient-reported outcome measures, *PROMIS* patient-reported outcomes measurement information system, *RA * rheumatoid arthritis, *RMDQ* roland morris disability questionnaire, *ROC* receiver operating characteristics, *SF-36* short form 36

^a^N reflects the number of patients on which the presented MIC values are based (often a subset of the study population)

^b^MIC values for minimal important improvement, unless otherwise specified. For all values, higher MIC values indicate more improvement or more deterioration for the construct being measured

**Table 2:** Minimal important change values for adult PROMIS physical function

References	PROMIS measure	Language	Population	Intervention	Method used	Follow-up	N^a^	Correlation of PROMIS change score with anchor	MIC value^b^	Comments
Bartlett et al. [78]	V1.0 Physical Function 20a	English (US)	Rheumatoid arthritis	Medication	Mean change of individuals responding a little better or a little worse on an anchor question on change in physical function	4.6 (2.4) Months	33	Not reported	Improvement: 2.8	
							46		Deterioration: 1.4	
							33		Improvement: 2.5	
	V1.0 physical function 4a						45		Deterioration: 1.8	
Bernstein et al. [47]	V1.0 physical function	English (US)	Carpal tunnel release	Surgery	Mean change in subgroup who reached a MIC on MHQ Function (23 point) or BCTQ (0.74 points)	6 Weeks or 3 months	52 (MHQ)	Not reported	1.8	MIC of MHQ Function was based on MHQ satisfaction, where ‘satisfied’ was defined based on effect size (distribution-based)
							40 (BCTQ)		2.8	
Hays et al. [51]	V1.0 physical function 19 (out of 20a)	English (US)	Rheumatoid arthritis	Any treatment	Mean change in patients who reported “a little better” or “a little worse” on an anchor question on how you are feeling now compared to 6 months ago	0–6 Months 6–12 months	35	0.33	Improvement: 1.6 2.0	Anchor question does not refer to change in physical function
						0–6 Months 6–12 months	113		Deterioration: 0.8 1.7	
Hung et al. [53]	V1.2 physical function CAT	English (US)	Foot and ankle disorders	Surgery	Optimal ROC cut-off point to distinguish patients who reported much worse, worse, improved and much improved from patients who reported slightly worse, no change, slightly improved on an anchor question on change in physical function	3 Months	32 vs 42 (43% changed)	Not reported	12.0	Patients who were improved and patients who were deteriorated were lumped together in the analyses
						> 3 Months	219 vs 337 (39% changed)		7.9	
						> 6 Months	128 vs 178 (42% changed)		10.5	
Hung et al. [54]	V1.2 physical function CAT	English (US)	Pathology of the hip or knee	Surgical and non-surgical interventions	Optimal ROC cut-off point to distinguish patients who reported much worse, worse, improved and much improved from patients who reported slightly worse, no change, slightly improved on an anchor question on change in physical function	3 Months	54 vs 88 (38% improved)	Not reported	2.0	
						> 3 Months	366 vs 577 (39% improved)		3.4	
						6 Months	21 vs 34 (38% improved)		3.5	
						> 6 Months	192 vs 421 (31% improved)		8.2	
Katz et al. [70]	Physical Function 4a	English (US)	SLE	Not reported	Mean change of patients who reported somewhat better on an anchor question on change in functioning	4 Periods of 6 months	141	Not reported	Improvement: 0.8	Each patient was included up to 4 times in the analysis
							169		Deterioration: 1.1	
Kazmers et al. [86]	V2.0 physical function CAT	English (US)	Non-shoulder hand and upper extremity pathology	Recovering from surgery, undergoing surgery, corticosteroid injection, other	Mean difference between patients reporting no change and patients reporting (slightly) improved on an anchor question (2 anchor questions)	6 (± 4) Weeks	381	Not reported	2.1, 3.6	
Kenney et al. [56]	Physical function CAT	English (US)	patients undergoing knee arthroscopy	knee arthroscopy	Optimal ROC cut-off point to distinguish patients who reached a MIC (11.5 points on 0–100 scale) on the IKDC	2 Weeks to 12 months	51 vs 25 (67% improved)	0.76	3.3	Version not reported
Khutok et al. [87]	Physical function 4a (part of PROMIS-29 v2.1)	Thai	Chronic low back pain	Many received standard physical therapy	Mean change of individuals reporting little improvement on an anchor question on change in pain interference	4 Weeks	54	0.26	0.1	
Kuhns et al. [88]	Physical function CAT	English (US)	Patients undergoing hip arthroscopy, (including 61 athletes)	Hip arthroscopy	Optimal ROC cut-off point to distinguish patients who reported their level of function after surgery excellent or good versus patients who reported their level of function after surgery average, fair or poor	21.3 (± 4.4) Months	113	Not reported	5.1	Anchor question about current (postoperative) level of functioning instead of change
Lapin et al. [59]	V1.0 physical function CAT	English (US)	Ischemic and hemorrhagic stroke patients	Routine care	Mean change in patients who indicated minimally or much improved/worse on an anchor on change in function	5–6 Months	167	0.28	Improvement: 4.0 (± 7.4)	Minimally and much improved patients were combined, MIC likely overestimated
							34		Deterioration: 0.3 (± 7.0)	
Lee et al. [60]	V1.0 physical function 10a	English (US)	Adults (40+) with knee OA	Tai Chi or physical therapy	Mean change in patients that reached 1–2 MICs on SF-36 PF	12 Weeks	13–28	0.59	1.9 to 2.2	Unclear which MIC values for SF-PF were used and whether they were anchor-based Lower bound of MICs was increased to > SEM
Lee et al. [76]	V1.2/V2.0 physical function CAT	English (US)	Thumb carpometacarpal arthritis	Operative or non-operative treatment	Mean change score in the mild improvement group according to an anchor question on change in your condition since last visit	63 days (IQR 42–125)	70	Not reported	3.9 (95% CI 3.3–4.7)	Anchor question does not refer to change in physical function
Sandvall et al. [64]	V1.2/V2.0 physical function CAT	English (US)	Unilateral distal radius fracture	Nonsurgical care	Mean change in patients who reported mildly better on an anchor question on change in your condition	Median 35 days (IQR, 25 − 45)	138	Not reported	3.6	Anchor question does not refer to change in physical function
Smit et al. [90]	24-item PROMIS physical function geriatric rehabilitation (PROMISPF-GR) short form	Dutch (NL)	patients admitted for geriatric inpatient rehabilitation	Usual care in nursing homes	predictive modeling to distinguish patients who reported to be improved versus patients who reported to be unchanged on an anchor question on change in physical function	41 Days	167	0.32	8.0 (95% CI 4.1–12.5)	
Speck et al. [91]	V1.0 physical function 13-item custom short form	English (US, Canada, EU, and Australia)	Tenosynovial giant cell tumors	pexidartinib or placebo	Mean change in patients who changed 1 point on a 5-point global rating of physical function	25 Weeks	27	Not reported	4.0	Design and sample size not clearly reported
Stephan et al. [99]	V2.0 physical function 4a	German (Switzerland)	Foot and ankle disorders	Orthopedic foot and ankle surgery	Optimal ROC cut-off point to distinguish patients who reported operation did help or operation helped a lot from patients who reported operation helped only a little, did not help or made things worse	6 Months	166 vs 36 (82% improved)	0.45	4.6	Anchor does not refer to change in physical function MIC overestimated due to high percentage of patients improved
Yost 2011 [45]	V1.1 cancer physical function-10	English (US)	Advanced-stage cancer	Chemotherapy only (74.3%) Chemo- and radiation therapy (9.9%) Other mixed modalities (13.8%) Missing 2.0%	Mean change in patients who changed 1–2 MICs on SF-36 PF (8–16 points) Mean change in patients who reported a little better or moderately better or a little worse or moderately worse	6–12 Weeks	Subgroups of 88	> 0.30	Median 3.0 (range 3.0–3.0)	PROMIS Cancer scales are on the same metric as the PROMIS generic item banks Estimates for improvement and deterioration were lumped together MIC of SF-36 partly based on distribution-based methods

*BCTQ*  boston carpal tunnel questionnaire, *CAT* computerized adaptive testing, *IKDC*  international knee documentation committee, *IQR* inter quartile range, *MIC* minimal important change, *MHQ* Michigan hand questionnaire, *NA* not applicable, *NRS* numerical rating scale, *OA*  osteoarthritis, *PROMIS* patient-reported outcomes measurement information system, *ROC* receiver operating characteristics, *SEM* standard error of measurement, *SF-36 PF* short form 36, physical function subscale

^a^N reflects the number of patients on which the presented MIC values are based (often a subset of the study population)

^b^MIC values for minimal important improvement, unless otherwise specified. For all values, higher MIC values indicate more improvement or more deterioration for the construct being measured

**Table 3:** Minimal important change values for adult PROMIS fatigue

References	PROMIS measure	Language	Population	Intervention	Method used	Follow-up	N^a^	Correlation of PROMIS change score with anchor	MIC value^b^	Comments
Bartlett et al. [78]	V1.0 fatigue 8a	English (US)	Rheumatoid arthritis	Medication	Mean change of individuals responding a little better or a little worse on an anchor question on change in fatigue	4.6 (2.4) months	37	Not reported	Improvement: 2.7	
							62		Deterioration: 1.3	
	V1.0 fatigue 7a						37		Improvement: 2.6	
							62		Deterioration: − 0.3	
	V1.0 fatigue 4a						37		Improvement: 3.3	
							60		Deterioration: 1.2	
Beaumont et al. [79]	V1.0 fatigue 7a	English (US)	Rheumatoid arthritis	Not reported	Mean change of individuals responding a little better or a little worse on an anchor question on change in fatigue	6 months	41	0.29	Improvement: 2.6	Correlation with anchor very low
							119		Deterioration: 1.7	
						12 months	31	0.13	Improvement: 1.3	
							133		Deterioration: 0.9	
Bingham et al. [80]	V1.0 fatigue	English (US)	Rheumatoid arthritis	NA	Qualitative bookmarking method	NA	11	NA	Improvement: 5	
									Deterioration: 10–15	
Katz et al. [70]	V1.0 fatigue 4a	English (US)	SLE	Not reported	Mean change of individuals responding somewhat better or somewhat worse on an anchor question on change in function	6 months	26	Not reported	Improvement: 2.3	
							57		Deterioration: 1.9	
Khutok et al. [87]	Fatigue 4a (part of PROMIS-29 v2.1)	Thai	Chronic low back pain	Many received standard physical therapy	Mean change of individuals reporting little improvement on an anchor question on change in pain intensity	4 weeks	45	0.13	1.9	
Lapin et al. [59]	V1.0 fatigue CAT	English (US)	Ischemic and hemorrhagic stroke patients	Routine care	Mean change in patients who indicated minimally or much improved/worse	5–6 months	140	0.27	Improvement: 2.4 (± 8.3)	Minimally and much improved patients were combined, MIC likely overestimated
							29		Deterioration: 3.0 (± 7.7)	
Yost et al. [45]	V1.0 cancer fatigue-17	English (US)	Advanced-stage cancer	Chemotherapy only (74.3%) Chemo- and radiation therapy (9.9%) Other mixed modalities (13.8%) Missing 2.0%	Mean change in patients who had a one point change on an anchor with range − 4 to 4	6–12 weeks	Subgroups of 88	> 0.30	Median 2.6 (range 1.9–3.6)	Cancer scales are on the same metric as the generic item banks
					Mean change in patients who changed 1–2 MICs on FACIT-Fatigue (3–6 points)					
	V1.0 cancer fatigue-7				Mean change in patients who reported a little better or moderately better or a little worse or moderately worse				Median 2.4 (range 1.9–4.2)	Estimates for improvement and deterioration were lumped together

*CAT*  computerized adaptive testing, *FACIT* functional assessment of chronic illness therapy, *MIC* minimal important change, *PROMIS* patient-reported outcomes measurement information system

^a^N reflects the number of patients on which the presented MIC values are based (often a subset of the study population)

^b^MIC values for minimal important improvement, unless otherwise specified. For all values, higher MIC values indicate more improvement or more deterioration for the construct being measured

Out of the 28 studies that used anchor-based methods 12 studies reported the correlation between the PROMIS change scores and the anchor. These correlations ranged from 0.02 to 0.76.

In several studies MIC values were presented for more than one PROMIS item bank. Regarding the adult PROMIS item banks, most MIC estimates were found for Pain Interference [17 studies, including 19 patient samples, MIC values for improvement ranged from 0.7 to 12.4 (Table 1)] and Physical Function [18 studies, MIC values for improvement ranged from 0.1 to 12.0 (Table 2)]. Multiple studies were found for Fatigue [7 studies, MIC values for improvement ranged from 1.3 to 5 (Table 3)], Anxiety [5 studies, MIC values for improvement ranged from 2.3 to 3.5 (Table S1 is found in Online Supplement 1)], Depression [4 studies, MIC values for improvement ranged from 1.5 to 3.7 (Table S2 is found in Online Supplement 1)], Upper Extremity [4 studies, MIC values for improvement ranged from 3.0 to 10.3 (Table S3 is found in Online Supplement 1)], Sleep Disturbance [3 studies, MIC values for improvement ranged from 0.9 to 2.4 (Table S4 is found in Online Supplement 1)], Ability to Participate in Social Roles and Activities [3 studies, MIC values for improvement ranged from 0.4 to 2.2 (Table S5 is found in Online Supplement 1)], and Pain Intensity [2 studies, MIC values for improvement ranged from 1.2 to 4.0 (Table S7 is found in Online Supplement 1)]. For the domains Satisfaction with Social Roles and Activities, Gastrointestinal Symptoms, Itch, and Global Health, only one study was found (Tables S6, S8, S9, S10, S11 is found in Online Supplement 1).

Only two studies estimated MIC values for five different PROMIS pediatric item banks (Mobility, Upper Extremity, Pain Interference, Fatigue, and Depressive Symptoms, Table S11), with MIC values ranging from 0.1 to 12.7 [41, 61].

## Discussion

We defined the minimal important change (MIC) as a threshold for a *minimal* within-person *change* over time above which patients perceive themselves *importantly* changed. Assuming that all patients have their individual threshold of what they consider a minimal important change, the MIC can be conceptualized as the mean of these individual thresholds. The MIC can be used to determine the number of responders in a group of patients to interpret study results or to inform patients about expected treatment results, or to help clinicians to estimate the probability that an individual patient has experienced a meaningful change, facilitating a conversation with the patient.

There is no perfect MIC method. Distribution-based methods are not appropriate because they do not relate to the importance of the change to patients. We consider the predictive modeling method the most appropriate anchor-based method, because, unlike the mean change method, it refers to a *threshold* for minimal important change. Moreover, the MIC_predict_ is more precise than the MIC_ROC_ and a formula has been published to correct the MIC_predict_ for bias if the percentage of improved patients is not 50% [20]. A disadvantage of all anchor-based MIC methods is the concern about the reliability and validity of the anchor question. The relatively new vignette-based method does not depend upon an anchor question, but the MIC_vignette_ may represent a value higher than a minimal threshold if based on mean differences between vignettes. We recommend the predictive modeling method, possibly supplemented with the vignette-based method if time and knowledge to design vignettes and recruit patients for that kind of study is available.

Our systematic review showed that published MIC estimates for PROMIS measures vary widely (larger than the range of MIC estimates currently published on the HealthMeasures website [93]) and were often generated by less appropriate methods. The lower end of the observed range of MIC values (0.1 T-score points) is, in our opinion, implausible as a MIC threshold. The highest MIC values (7 T scores points of higher) were almost all found in adult patients undergoing surgery. It has been suggested before that an invasive procedure like surgery might require a higher change to be considered an important improvement, but results in the literature have been inconsistent [94, 95]. For non-surgical interventions, we consider a MIC value of 2–6 points (covering about two thirds of the published MIC values) reasonable to assume at this point. There is not enough evidence yet to make more specific domain-specific or population-specific recommendations. Further studies are needed to examine whether MIC values differ across domains or between adults and children.

**Fig. 1:**
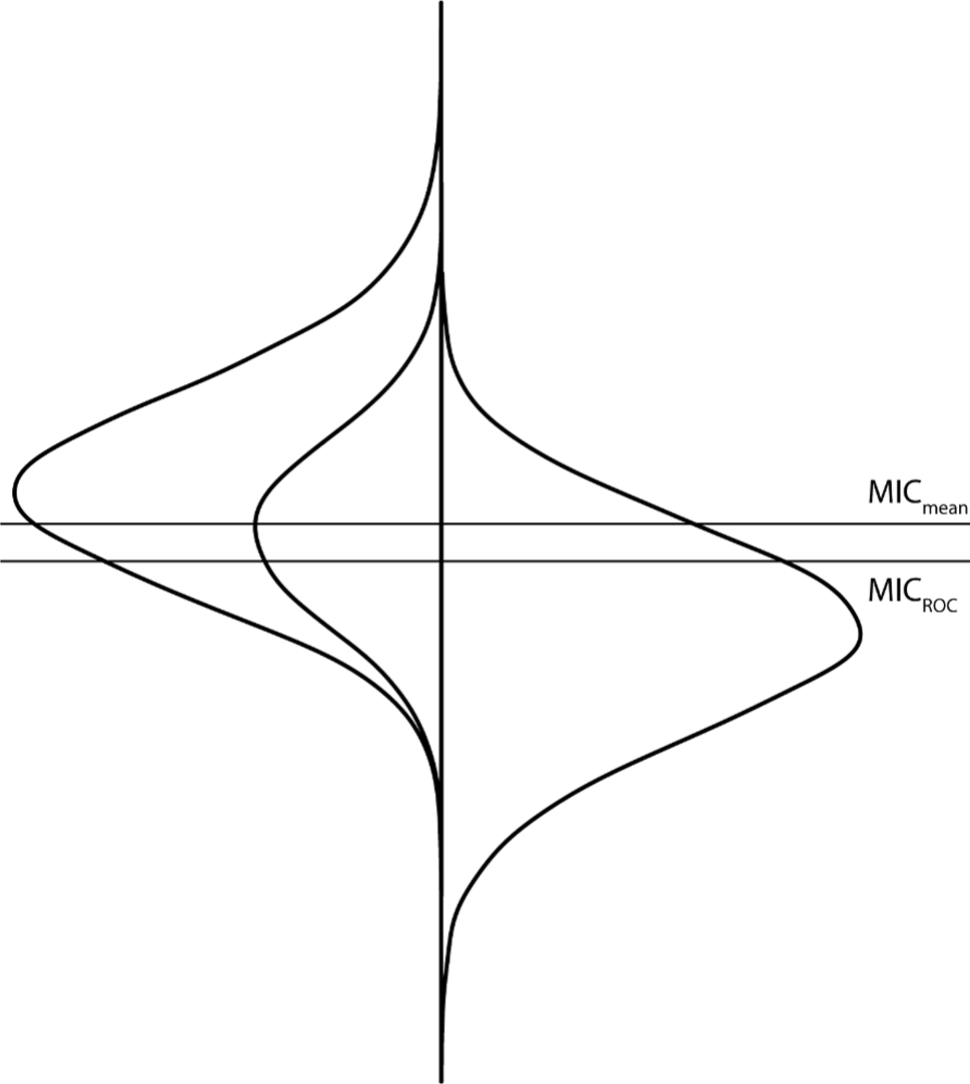
MIC_mean_ and MIC_ROC_. On the left, the distribution of change scores in all patients who are ‘improved’ (larger distribution) and in patients who are ‘a little better’ (smaller distribution), on the right the distribution of change scores in patients who are ‘not improved’. The upper line represents the MIC_mean_ (based on the smaller distribution on the left side), the lower line represents the MIC_ROC_ (based on the larger left-sided distribution and the right-sided distribution)

We particularly noticed several methodological concerns which might result in such a wide range of MIC estimates. First, most of these studies used the mean change method, which may represent a value higher than a minimal threshold. We did not exclude these results because this method is currently the most widely used method in the field (despite the critiques raised here) and only five studies used the ROC method, one study used the predictive modeling method [90], and three studies used a vignette-based method. In theory, it is likely that MIC_mean_ values represent an overestimation of the MIC (Fig. 1); however, many reported MIC values were rather low. Second, sample sizes on which the MIC estimates were based were often small. Third, some studies used the MIC of another instrument as an anchor. These MIC values were sometimes untraceable, based on the MIC value of yet another instrument, based on instruments that may not measure a sufficiently-similar construct or that lack evidence for responsiveness, or based on distribution-based methods. Fourth, only 12 out of 28 anchor-based studies presented the correlation between the PROMIS change score and the anchor question and about one third of the correlations were lower than 0.30 (excluding these values would not change our conclusions). Fifth, in some studies it was not clear whether the MIC estimate was based on patients who improved minimally. Sixth, in some studies the lower bound of recommended MIC values was increased to the SEM. However, the SEM represents the amount of measurement error and does not reflect changes that patients consider important. For this reason, setting a MIC lower bound to be in the detectable range may eliminate changes that patients find important. More broadly, researchers should be mindful of instruments with large measurement error and attempt to reduce the measurement error (e.g., using CAT), instead of adjusting the MIC value [3]. Finally, in some studies improved and deteriorated patients were combined together, while the MIC for improvement might be different than the MIC for deterioration, making inferences about the estimated MIC difficult [31, 33–35].

Another problem is that important details of the MIC studies were often not reported, such as version numbers (while different versions of PROMIS measures may have a different metric), percentage of patients improved, correlation between the PROMIS change score and the anchor, and samples size on which MIC value was based. Recently, a reporting guideline for all publications using PROMIS and other HealthMeasures instruments was published [96]. We strongly recommend PROMIS users to use these reporting recommendations. A reporting guideline for MIC studies is being developed by an international group led by researchers from McMaster University, Canada (personal communication).

To gain more insight in the meaning of PROMIS change scores, more high-quality MIC studies are needed. To increase the understanding of the concept of MIC and improve the field, we need to agree on a clear definition of the MIC and report MIC values that are based on this definition. We recommend not to publish MIC values based on data where the correlation between the change score and the anchor is too low. We recommend to report the anchor correlations and state that the low correlation prevents MIC estimation, rather than publish MIC values based on distribution-based methods. We offer recommendations for conducting MIC studies (Box 1) that may help preventing the situation where the correlation between the change score and the anchor is too low. Alternatively, we recommend to use vignette-based methods. The recommendations in Box 1 can also be used to re-analyze existing data. More data are also needed to examine whether the MIC value differs across the PROMIS metric and across settings (e.g., duration of disease, kind of intervention, length of follow-up) [26]. In case researchers need to analyze a study (e.g., responders in a clinical trial) and no credible anchor-based MIC value is available, researchers could decide to use a distribution-based value, such as 0.5 × SD, or use a range of different values in a sensitivity analysis, but we argue that these values should not be called MIC values because distribution-based values refer to the concept of measurement error and are not based on the concept of MIC. However, as stated in part 1, researchers should keep in mind that the estimated MIC value is derived from a wider sample of patients, and the MIC threshold or responder classification may not apply to the individual patient in the clinical trial or in the consultation room.

This study has some limitations. First, we only searched PubMed and the abstracts were screened by one author only, so we may have missed some MIC studies. Second, we based our review on one definition of minimal important change and excluded studies and MIC estimates that were not in line with this definition. Others may have different opinions, and the excluded studies and estimates may nevertheless provide relevant information about the interpretation of PROMIS (change) scores. Strong points of the study were that data extraction was checked by a second author and missing information was requested by email from the corresponding authors of the papers.

In conclusion, 50 studies estimated the MIC of a PROMIS measure, of which 19 studies used less appropriate methods. MIC values of the remaining 31 studies ranged from 0.1 to 12.7 T scores points. We consider a MIC value of 2–6 T-score points for PROMIS measures reasonable to assume at this point. For surgical interventions a higher MIC value might be appropriate. We recommend more high-quality studies estimating MIC values for PROMIS. This paper provides recommendations for designing and analyzing future MIC studies.

## Supplementary Information

Below is the link to the electronic supplementary material.

Supplementary file1 (DOCX 132 kb)
