# Supplementary material for: Minimal important change (MIC): a conceptual clarification and systematic review of MIC estimates of PROMIS measures
Source: Qual Life Res. 2021 Jul 10;30(10):2729–54. doi: 10.1007/s11136-021-02925-y (PMC8481206; doi:10.1007/s11136-021-02925-y)
Supplement: Supplementary file 1 — Supplementary file1 (DOCX 132 kb) [file 11136_2021_2925_MOESM1_ESM.docx]

**Online supplement 1**

This supplement belongs to the paper:

**Minimal Important Change (MIC): A conceptual clarification and systematic review of MIC estimates of PROMIS measures**

Caroline B Terwee, John Devin Peipert, Robert Chapman, Jin-Shei Lai, Berend Terluin, David Cella, Pip Griffith, Lidwine B Mokkink

**Table S1. Minimal Important Change values for adult PROMIS Anxiety**

| **Ref** | **PROMIS measure** | **Language** | **population** | **intervention** | **Method used** | **Follow-up** | **N^1^** | **Correlation of PROMIS change score with anchor** | **MIC value^2^** | **Comments** |
| --- | --- | --- | --- | --- | --- | --- | --- | --- | --- | --- |
| Bartlett 2021 [77] | V1.0 Anxiety 4a | English (US) | Rheumatoid arthritis | Medication | Mean change of individuals responding a little better or a little worse on an anchor question on change in rheumatoid arthritis. | 4.6 (2.4) months | 43  60 | Not reported | Improvement: 2.4  Deterioration: 1.1 |  |
| Khutok 2021 [86] | Anxiety 4a (part of PROMIS-29 v2.1) | Thai | Chronic low back pain | Many received standard physical therapy | Mean change of individuals reporting little improvement on an anchor question on change in pain intensity | 4 weeks | 47 | 0.34 | 3.4 |  |
| Lapin 2019 [58] | v1.0 Anxiety CAT | English (US) | Ischemic and hemorrhagic stroke patients | Routine care | Mean change in patients who indicated  minimally or much improved/worse. | 5-6 months | 49 | 0.26 | Improvement:  3.5 (± 10.0) | Minimally and much improved patients were combined, MIC likely overestimated |
| Lee 2017 [59] | V1.0 Anxiety 7a | English (US) | Adults (40+) with Knee OA | Tai Chi or physical therapy | Mean change in patients that reached 1-2 MICs on SF-36 subscale | 12 weeks | 20-42 | >0.30 | 2.3 to 3.4 | Unclear which MIC values for SF-36 were used and whether they were anchor-based.  Lower bound of the MIC range was set to the SEM |
| Yost 2011 [44] | V1.0 Cancer Anxiety-9 | English (US) | Advanced-stage cancer | Chemotherapy only (74.3%)  Chemo- and radiation therapy (9.9%)  Other mixed modalities (13.8%)  Missing 2.0% | Mean change in patients who changed 1-2 MICs on HADS (1.5-3 points)  Mean change in patients who reported a little better or moderately better or a little worse or moderately worse | 6-12 weeks | Subgroups of 88 | >0.30 | Median 3.1  (range 1.6-4.7) | Cancer scales are on the same metric as the generic item banks  Estimates for improvement and deterioration were lumped together  Lower bound of MICs was increased to >SEM |

^1^ N reflects the number of patients on which the presented MIC values are based (often a subset of the study population)

^2^ MIC values for minimal important improvement, unless otherwise specified. For all values, higher MIC values indicate more improvement or more deterioration for the construct being measured.

CAT = Computerized Adaptive Testing; HADS= Hospital Anxiety and Depression Scale; MIC = Minimal Important Change; OA = Osteoarthritis; PROMIS = Patient-Reported Outcomes Measurement Information System; SEM = Standard Error of Measurement; SF-36 = Short Form 36.

**Table S2. Minimal Important Change values for adult PROMIS Depression**

| **Ref** | **PROMIS measure** | **Language** | **population** | **intervention** | **Method used** | **Follow-up** | **N^1^** | **Correlation of PROMIS change score with anchor** | **MIC value^2^** | **Comments** |
| --- | --- | --- | --- | --- | --- | --- | --- | --- | --- | --- |
| Bartlett 2021 [77] | V1.0 Depression 4a | English (US) | Rheumatoid arthritis | Medication | Mean change of individuals responding a little better on an anchor question on change in rheumatoid arthritis. | 4.6 (2.4) months | 43  60 | Not reported | Improvement: 1.5  Deterioration: 1.5 |  |
| Khutok 2021 [86] | Depression 4a (part of PROMIS-29 v2.1) | Thai | Chronic low back pain | Many received standard physical therapy | Mean change of individuals reporting little improvement on an anchor question on change in pain intensity | 4 weeks | 34 | 0.16 | 1.5 |  |
| Lee 2017 [59] | V1.0 Depression 8b | English (US) | Adults (40+) with Knee OA | Tai Chi or physical therapy | Mean change in patients that reached 1-2 MICs on SF-36 subscale | 12 weeks | 20-42 | >0.30 | 3.0 to 3.1 | Unclear which MIC values for SF-36 were used and whether they were anchor-based.  Lower bound of the MIC range was set to the SEM |
| Yost 2011 [44] | V1.0 Cancer Depression-10 | English (US) | Advanced-stage cancer | Chemotherapy only (74.3%)  Chemo- and radiation therapy (9.9%)  Other mixed modalities (13.8%)  Missing 2.0% | Mean change in patients who changed 1-2 MICs on HADS (1.5-3 points)  Mean change in patients who reported a little better or moderately better or a little worse or moderately worse | 6-12 weeks | Subgroups of 88 | >0.30 | Median 2.7  (range 2.1-3.7) | PROMIS Cancer scales are on the same metric as the PROMIS generic item banks  Estimates for improvement and deterioration were lumped together  Lower bound of MICs was increased to >SEM |

^1^ N reflects the number of patients on which the presented MIC values are based (often a subset of the study population)

^2^ MIC values for minimal important improvement, unless otherwise specified. For all values, higher MIC values indicate more improvement or more deterioration for the construct being measured.

CAT = Computerized Adaptive Testing; HADS= Hospital Anxiety and Depression Scale; MIC = Minimal Important Change; OA = Osteoarthritis; PROMIS = Patient-Reported Outcomes Measurement Information System; SEM = Standard Error of Measurement; SF-36 = Short Form 36.

**Table S3. Minimal Important Change values for adult PROMIS Upper Extremity**

| **Ref** | **PROMIS measure** | **Language** | **population** | **intervention** | **Method used** | **Follow-up** | **N^1^** | **Correlation of PROMIS change score with anchor** | **MIC value^2^** | **Comments** |
| --- | --- | --- | --- | --- | --- | --- | --- | --- | --- | --- |
| Bernstein 2019 [46] | V1.2 Upper Extremity CAT | English (US) | Carpal tunnel release | Surgery | Mean change in subgroup that reached an MIC on MHQ (23 points) or BCTQ (0.74 points) | 6 weeks or 3 months | 52 (MHQ) | No reported | 6.3 | MIC of MHQ was based on MHQ satisfaction, where ‘satisfied’ was defined based on effect size (distribution-based) |
|  |  |  |  |  |  |  | 40 (BCTQ) |  | 8.0 |  |
| Forlenza 2021 [80] | Upper Extremity CAT | English (US) | Biceps tenodesis | Surgery | Optimal ROC cut-off point to distinguish patients who reported improvement from patients who reported no improvement on an anchor question on overall function of the shoulder | 7.6 (6.0-9.3) months | 112 | Not reported | 10.3 |  |
| Kazmers 2021 [84] | V1.2 Upper Extremity CAT | English (US) | thumb carpometacarpal  osteoarthritis | Hand surgery | Mean change in patients reporting little relief /improvement as a results of treatment on an anchor question | 2-24 weeks | 145 | Not reported | 4.2 |  |
| Kazmers 2021 [85] | V2.0 Upper Extremity CAT | English (US) | Non-shoulder hand and upper extremity pathology | Recovering from surgery, undergoing surgery, corticosteroid injection, other | Mean difference between patients reporting no change and patients reporting (slightly) improved on an anchor question (2 anchor questions) | 6 (± 4) weeks | 381 | Not reported | 3.0, 4.0 |  |

^1^ N reflects the number of patients on which the presented MIC values are based (often a subset of the study population)

^2^ MIC values for minimal important improvement, unless otherwise specified. For all values, higher MIC values indicate more improvement or more deterioration for the construct being measured.

BCTQ = Boston Carpal Tunnel Questionnaire; MHQ = Michigan Hand Questionnaire; MIC = Minimal Important Change; PROMIS = Patient-Reported Outcomes Measurement Information System.

**Table S4. Minimal Important Change values for adult PROMIS Sleep Disturbance**

| **Ref** | **PROMIS measure** | **Language** | **population** | **intervention** | **Method used** | **Follow-up** | **N^1^** | **Correlation of PROMIS change score with anchor** | **MIC value^2^** | **Comments** |
| --- | --- | --- | --- | --- | --- | --- | --- | --- | --- | --- |
| Bartlett 2021 [77] | V1.0 Sleep Disturbance 4a | English (US) | Rheumatoid arthritis | Medication | Mean change of individuals responding a little better or a little worse on an anchor question on change in rheumatoid arthritis. | 4.6 (2.4) months | 43  60 | Not reported | Improvement: 1.6  Deterioration: -1.6 |  |
| Katz 2020 [69] | V1.0 Sleep Disturbance 4a | English (US) | SLE | Not reported | Mean change of individuals responding somewhat better or somewhat worse on an anchor question on change in function | 6 months | 25 | Not reported | Improvement: 2.4 |  |
|  |  |  |  |  |  |  | 51 |  | Deterioration: 2.5 |  |
| Khutok 2021 [86] | Sleep Disturbance 4a (part of PROMIS-29 v2.1) | Thai | Chronic low back pain | Many received standard physical therapy | Mean change of individuals reporting little improvement on an anchor question on change in pain intensity | 4 weeks | 39 | 0.16 | 0.9 |  |

^1^ N reflects the number of patients on which the presented MIC values are based (often a subset of the study population)

^2^ MIC values for minimal important improvement, unless otherwise specified. For all values, higher MIC values indicate more improvement or more deterioration for the construct being measured.

CAT = Computerized Adaptive Testing; MIC = Minimal Important Change; PROMIS = Patient-Reported Outcomes Measurement Information System.

**Table S5. Minimal Important Change values for adult PROMIS Ability to Participate in Social Roles and Activities**

| **Ref** | **PROMIS measure** | **Language** | **population** | **intervention** | **Method used** | **Follow-up** | **N^1^** | **Correlation of PROMIS change score with anchor** | **MIC value^2^** | **Comments** |
| --- | --- | --- | --- | --- | --- | --- | --- | --- | --- | --- |
| Bartlett 2021 [77] | V2.0 Ability to Participate in Social Roles and Activities 8a  V2.0 Ability to Participate in Social Roles and Activities 4a | English (US) | Rheumatoid arthritis | Medication | Mean change of individuals responding a little better on an anchor question on change in pain interference. | 4.6 (2.4) months | 36  35 | Not reported | 2.2  2.2 |  |
| Katz 2020 [69] | V2.0 Ability to Participate in Social Roles and Activities 4a | English (US) | SLE | Not reported | Mean change of individuals responding somewhat better or somewhat worse on an anchor question on change in function | 6 months | 25 | Not reported | Improvement: 0.4 |  |
|  |  |  |  |  |  |  | 36 |  | Deterioration: 2.2 |  |
| Khutok 2021 [86] | Ability to Participate in Social Roles and Activities 4a (part of PROMIS-29 v2.1) | Thai | Chronic low back pain | Many received standard physical therapy | Mean change of individuals reporting little improvement on an anchor question on change in pain intensity | 4 weeks | 39 | 0.02 | 0.5 |  |

^1^ N reflects the number of patients on which the presented MIC values are based (often a subset of the study population)

^2^ MIC values for minimal important improvement, unless otherwise specified. For all values, higher MIC values indicate more improvement or more deterioration for the construct being measured. SLE = Systemic Lupus Erythematosus

**Table S6. Minimal Important Change values for adult PROMIS Satisfaction with Social Roles and Activities**

| **Ref** | **PROMIS measure** | **Language** | **population** | **intervention** | **Method used** | **Follow-up** | **N^1^** | **Correlation of PROMIS change score with anchor** | **MIC value^2^** | **Comments** |
| --- | --- | --- | --- | --- | --- | --- | --- | --- | --- | --- |
| Lapin 2019 [58] | V1.0 Satisfaction  with social roles CAT | English (US) | Ischemic and hemorrhagic stroke patients | Routine care | Mean change in patients who indicated  minimally or much improved/worse. | 5-6 months | 48 | 0.33 | Improvement:  6.2 (± 8.5) | Minimally and much improved patients were combined, MIC likely overestimated |

^1^ N reflects the number of patients on which the presented MIC values are based (often a subset of the study population)

^2^ MIC values for minimal important improvement, unless otherwise specified. For all values, higher MIC values indicate more improvement or more deterioration for the construct being measured.

CAT = Computerized Adaptive Testing; MIC = Minimal Important Change; PROMIS = Patient-Reported Outcomes Measurement Information System.

**Table S7. Minimal Important Change values for adult PROMIS Pain Intensity**

| **Ref** | **PROMIS measure** | **Language** | **population** | **intervention** | **Method used** | **Follow-up** | **N^1^** | **Correlation of PROMIS change score with anchor** | **MIC value^2^** | **Comments** |
| --- | --- | --- | --- | --- | --- | --- | --- | --- | --- | --- |
| Khutok 2021 [86] | Pain Intensity (0-10, part of PROMIS-29 v2.1) | Thai | Chronic low back pain | Many received standard physical therapy | Mean change of individuals reporting little improvement on an anchor question on change in pain intensity | 4 weeks | 54 | 0.25 | 1.2 |  |
| Stephan 2019 [98] | V1.0 Pain Intensity 3a | German (Switzerland) | Foot and ankle disorders | Orthopedic foot and ankle surgery | Optimal ROC cut-off point to distinguish patients who reported operation did help or operation helped a lot from patients who reported operation helped only a little, did not help or made things worse | 6 months | 166 vs 36  (82% improved) | 0.45 | 4.0 | Anchor does not refer to change in physical function  MIC overestimated due to high percentage of patients improved |

^1^ N reflects the number of patients on which the presented MIC values are based (often a subset of the study population)

^2^ MIC values for minimal important improvement, unless otherwise specified. For all values, higher MIC values indicate more improvement or more deterioration for the construct being measured.

CAT = Computerized Adaptive Testing; MIC = Minimal Important Change; PROMIS = Patient-Reported Outcomes Measurement Information System; ROC = Receiver Operating Characteristics.

**Table S8. Minimal Important Change values for adult PROMIS Gastrointestinal Symptoms**

| **Ref** | **PROMIS measure** | **Language** | **population** | **intervention** | **Method used** | **Follow-up** | **N^1^** | **Correlation of PROMIS change score with anchor** | **MIC value^2^** | **Comments** |
| --- | --- | --- | --- | --- | --- | --- | --- | --- | --- | --- |
| Khanna 2017 [56] | PROMIS GI Symptoms | English (US) | Gastrointestinal  disorders | New treatment  or increase  or decrease in pharmacologic or non-pharmacologic  therapies | Mean change in patients who reported “somewhat better” or “somewhat worse” on GSRS | 88 (4–257) days | 23  30  28  36  42 | Reflux: 0.40  Diarrhea: 0.57  Constipation: 0.54  Belly pain: 0.48  Gas/bloat/flatulence: 0.51 | Improvement:  Reflux: 5  Diarrhea: 5  Constipation: 5  Belly pain: 6  Gas/bloat/flatulence: 6 | Original version, prior to V1.0 |
|  |  |  |  |  |  |  | 32  42  30  26  27 |  | Deterioration:  Reflux: 6  Diarrhea: 6  Constipation: 6  Belly pain: 6  Gas/bloat/flatulence: 6 |  |

^1^ N reflects the number of patients on which the presented MIC values are based (often a subset of the study population)

^2^ MIC values for minimal important improvement, unless otherwise specified. For all values, higher MIC values indicate more improvement or more deterioration for the construct being measured.

CAT = Computerized Adaptive Testing; GI = Gastrointestinal; GSRS = Gastrointestinal Symptom Rating Scale; MIC = Minimal Important Change; PROMIS = Patient-Reported Outcomes Measurement Information System.

**Table S9. Minimal Important Change values for adult PROMIS Itch**

| **Ref** | **PROMIS measure** | **Language** | **population** | **intervention** | **Method used** | **Follow-up** | **N^1^** | **Correlation of PROMIS change score with anchor** | **MIC value^2^** | **Comments** |
| --- | --- | --- | --- | --- | --- | --- | --- | --- | --- | --- |
| Silverberg 2021 [88] | PROMIS Itch (PIQ)  Itch interference SF8  Mood and Sleep SF8  Clothing and Physical Activity SF8  Scratching Behaviour SF4 | English (US) | Dermatology | Practice based | Mean change in patients with a 1-point improvement in patient-reported  global atopic dermatitis severity rating (0-4)  Mean change in patients with a 1-point improvement in Verbal Rating Scale (VRS) for worst itch (0-4) | ? | Global: 102  VRS: 73 | Not reported | Itch interference SF8: 2.4  Mood and Sleep SF8: 3.7  Clothing and Physical Activity SF8: 2.0  Scratching Behaviour SF4: 3.7  Itch interference SF8: 2.2  Mood and Sleep SF8: 4.8  Clothing and Physical Activity SF8: 3.6  Scratching Behaviour SF4: 3.4 |  |

^1^ N reflects the number of patients on which the presented MIC values are based (often a subset of the study population)

^2^ MIC values for minimal important improvement, unless otherwise specified. For all values, higher MIC values indicate more improvement or more deterioration for the construct being measured.

**Table S10. Minimal Important Change values for adult PROMIS Global Physical Health**

| **Ref** | **PROMIS measure** | **Language** | **population** | **intervention** | **Method used** | **Follow-up** | **N^1^** | **Correlation of PROMIS change score with anchor** | **MIC value^2^** | **Comments** |
| --- | --- | --- | --- | --- | --- | --- | --- | --- | --- | --- |
| Khalil 2020 [70] | V1.1 PROMIS Global Health – Physical Health subscale | English (US) | patients undergoing primary unilateral TKA | TKA | Optimal ROC cut-off points to distinguish patients who reached an MIC (6.8) on the KOOS-JR | 1 month  3 months  6 months  1 year | 699  170  134  57 | 0.40  0.38  0.47  0.54 | 2.5 | MIC value of the KOOS-JR was distribution-based (0.5*SD)  Each patient was included in the analysis multiple times |

^1^ N reflects the number of patients on which the presented MIC values are based (often a subset of the study population)

^2^ MIC values for minimal important improvement, unless otherwise specified. For all values, higher MIC values indicate more improvement or more deterioration for the construct being measured.

TKA = Total Knee Arthroplasy

**Table S11. Mincimal Important Change values for PROMIS pediatric measures**

| **Ref** | **PROMIS measure** | **Language** | **population** | **intervention** | **Method used** | **Follow-up** | **N^1^** | **MIC value^2^** | **Comments** |
| --- | --- | --- | --- | --- | --- | --- | --- | --- | --- |
| Morgan 2017 [60] | V1.0 Pediatric Mobility | English (US) | Adolescents with JIA, parents of JIA  patients, and clinicians who treat children with JIA. | NA | Qualitative modified bookmarking method | NA | 16 | Adolescents: 0.1-5.0  Parents: 1.3-5.4  Clinicians: 0.1-2.2 |  |
| Morgan 2017 [60] | V1.0 Pediatric  Upper Extremity | English (US) | Adolescents with JIA, parents of JIA  patients, and clinicians who treat children with JIA. | NA | Qualitative modified bookmarking method | NA | 16 | Adolescents: 0.1-3.1  Parents: 2.1-3.6  Clinicians: 0.4-1.8 |  |
| Morgan 2017 [60] | V1.0 Pediatric  Pain Interference | English (US) | Adolescents with JIA, parents of JIA  patients, and clinicians who treat children with JIA. | NA | Qualitative modified bookmarking method | NA | 16 | Adolescents: 3.3-7.6  Parents: 5.5-12.7  Clinicians: 2.1-5.3 |  |
| Morgan 2017 [60] | V1.0 Pediatric  measures Fatigue | English (US) | Adolescents with JIA, parents of JIA  patients, and clinicians who treat children with JIA. | NA | Qualitative modified bookmarking method | NA | 16 | Adolescents: 3.7-5.4  Parents: 3.5-9.4  Clinicians: 1.4-3.0 |  |
| Thissen 2016 [40] | V1.0/2.0 Pediatric  Depressive Symptoms Pain Interference Fatigue  Mobility | English (US) | Clinicians, parents, and adolescents; coming from four clinical sites | NA | Scale judgment  method vignettes  MIC=scale score difference between vignettes  corresponding to a probability of 0.5 that the pair is judged different | NA | 83 clinicians  68 adolescents  76 parents | Wrong-direction  responses omitted,:  2.1 (SE 0.6)  2.3 (SE 0.6)  2.4 (SE 0.7) | 4 item banks have been lumped together |
|  |  |  |  |  |  |  | 83 clinicians  68 adolescents  76 parents | Wrong-direction responses  Reversed  1.9 (SE 0.6)  2.1 (SE 0.6)  2.2 (SE 0.7) |  |

^1^ N reflects the number of patients on which the presented MIC values are based (often a subset of the study population)

^2^ MIC values for minimal important improvement, unless otherwise specified. For all values, higher MIC values indicate more improvement or more deterioration for the construct being measured.

CAT = Computerized Adaptive Testing; JIA = Juvenile Idiopathic Arthritis; MIC = Minimal Important Change; PROMIS = Patient-Reported Outcomes Measurement Information System.

**Online supplement 2**

This supplement belongs to the paper:

**Minimal Important Change (MIC): A conceptual clarification and systematic review of MIC estimates of PROMIS measures**

Caroline B Terwee, John Devin Peipert, Robert Chapman, Jin-Shei Lai, Berend Terluin, David Cella, Pip Griffith, Lidwine B Mokkink

**Guidance for calculating the MIC_predict_ and MIC_ROC_**

In this supplement we provide calculations for determining the MIC_predict_ and the MIC_ROC_. SPSS syntax and R codes are also provided. Before determining the MIC value, we recommend to calculate the correlation between the anchor question and the PROMIS change score. If the correlation is below 0.30, we recommend not to determine the MIC value based on this data because of possible lack of validity of the anchor [2]. If you use the MIC_predict_, please cite the paper by Terluin et al. [21].

**Procedure to determine MIC_predict_**

The aim of this analysis is to find the PROMIS change score that optimally predicts the probability of belonging to the improved (or deteriorated) group (according to the anchor), given the PROMIS change score. The MIC_predict_ is equal to the change score that is associated with a likelihood ratio (LR) of 1, which is the change score where the posttest probability (P_post_) of belonging to the improved group is equal to the pretest probability (P_pre_) of belonging to the improved group (Formula 1) [21]. Posttest refers to the situation when the change score is known (i.e. after completion of the follow-up PROM), and pretest refers to the situation *before* the change score is known.

Formula 1. $LR= \frac{{odds}_{post}}{{odds}_{pre}}=\frac{p_{post}}{p_{pre}}=1$

Step 1. Divide the study sample in two groups: (1) patients who reported ‘a little better’ or more on the anchor (the ‘improved’ group) and (2) patients who reported to be ‘not changed’, ‘a little worse’ or more on the anchor (the ‘not improved’ group). When estimating a MIC for deterioration the study sample is split into patients who reported to be ‘a little worse’ or more (the ‘deteriorated’ group) and patients who reported ‘not changed’, ‘a little better’ or more on the anchor (the ‘not deteriorated’ group).

Step 2. Perform a logistic regression analysis with the dichotomous group variable (improved versus not improved based on the anchor) as the outcome and the PROMIS change score as the determinant. The logistic regression model is presented in Formula 2.

Formula 2. $\log{(odds}_{post})=c+ b*X$

The ${odds}_{post}$ represents the posttest odds of being improved given a certain PROMIS change score (X). The odds equals the probability of belonging to the improved group given the PROMIS change score (p_post_), divided by the complement of this probability, which is the probability of belonging to the not improved group, given the PROMIS change score (i.e., ${Odds}_{post}= \frac{p_{post}}{1-p_{post}}$). The component *c* in Formula 2 refers to the intercept and $b$ refers to the regression coefficient.

Step 3. Calculate the MIC_predict_ using Formula 3.

Formula 3. ${MIC}_{predict}=(\log\left( {Odds}_{pre} \right)-c)/b$

Terluin et al. have provided an Excel worksheet to calculate MIC_predict_ and its 95% CI based on the percentage improved patients and the regression coefficients with their associated standard errors from Formula 2 [99].

Step 4. (only required when the percentage of improved patients is not 50%). If the percentage of improved patients (p_pre_) is larger than 50% the MIC_predict_ will be overestimated. When the percentage of improved patients is smaller than 50%, the MIC_predict_ will be underestimated. This is also the case for the MIC_ROC_ but for the MIC_predict_ a formula was developed to adjust the MIC_predict_. For more details, we refer to Terluin et al. [20].

Formula 4. ${MIC}_{predict(adjusted)}={MIC}_{predict}-\left( 0.090+0.103* Cor \right)* {SD}_{change}* log({odds}_{pre})$

Where MIC_predict(adjusted)_ = MIC_predict_, adjusted for the proportion improved patients; Cor = correlation between the PROMIS change score and the anchor; SD_change_ = standard deviation of the PROMIS change score.

Calculating MIC_pred_ with SPSS and Excel

In this syntax it is assumed that the group variable is coded as 0=not improved, 1=improved.

The analysis can be found in the menu under ”Analyze”, “Regression”, “Binary Logistic”.

LOGISTIC REGRESSION VARIABLES group

/METHOD=ENTER TScore_change

/PRINT=CORR.

Use the Excel worksheet provided by Terluin et al. to calculate MIC_predict_ (X for which LR=1) and its 95% CI from the regression coefficients and their standard errors [99].

Calculating MIC_ROC_ and MIC_pred_ with R

### Estimate MIC(ROC), predictive MIC and adjusted MIC ###

### with bootstrap-based confidence intervals ###

### Own data preparation

# You need a dataframe with 2 variables:

# One variable represents the change score, name it 'TScore_change',

# take care that positive change scores represent improvement.

# The other variable represents the anchor, name it 'group'.

# The anchor variable should be coded '1' for improved and '0' for not-improved.

# If the file is a text file, you can read the file into R using the following command:

dat <- read.table(file.choose(), header=T)

# This command opens a Windows dialogue box that allows you to browse to your file, en open it in R.

### Simulated data for demonstration

TScore_change <- rnorm(2000,10,10)

R <- 0.5

help <- R * TScore_change + sqrt(1-R^2) * rnorm(2000,10,10)

cor(TScore_change, help)

mean(TScore_change)

sd(TScore_change)

mean(help)

sd(help)

group <- numeric(2000)

group[help > 8] <- 1

mean(group)

dat <- data.frame(TScore_change, group)

### End of simulated data

### ROC analysis

library(pROC)

rocobj <- roc(group ~ TScore_change, data = dat, quiet = TRUE)

cuty <- coords(rocobj, x="best", input="threshold", ret="threshold",

best.method="youden", transpose = TRUE)

( mic.roc.boot <- cuty ) # MIC(ROC-Youden)

### Do logistic regression and calculate MIC(pred)

( prev <- mean(dat$group) ) # proportion improved

logods <- log(prev/(1-prev))

fit <- glm(group ~ TScore_change, data = dat, family = "binomial")

# summary(fit)

C <- coef(fit)[1] # intercept coefficient C

B <- coef(fit)[2] # regression coefficient B

mic.pred <- (logods-C)/B

print(unname(mic.pred)) # Predictive MIC

### MIC adjusted

( SD <- sd(dat$TScore_change) ) # SD of the change score

( Cor <- cor(dat$TScore_change, dat$group) ) # correlation anchor-change score

Scf <- 0.09 * SD + 0.103 * SD * Cor # S-coefficient

mic.adj <- mic.pred - Scf * logods

print(unname(mic.adj)) # Adjusted MIC

### BOOTSTRAPPING

ns <- 1000 # NR OF BOOTSTRAP SAMPLES

mic.pred.boot <- numeric(ns)

mic.adj.boot <- numeric(ns)

mic.roc.boot <- numeric(ns)

for(i in 1:ns) {

btsmp <- dat[sample(1:dim(dat)[1], nrow(dat), replace=TRUE),]

# prevent continuation with 0% or 100% improved cases

for(j in 1:10) {

if(mean(btsmp$group)==0 | mean(btsmp$group)==1) {

btsmp <- dat[sample(1:dim(dat)[1], nrow(dat), replace=TRUE),]

}

}

( Cor <- cor(btsmp$TScore_change, btsmp$group) )

( SD <- sd(btsmp$TScore_change) )

( prev <- mean(btsmp$group) )

( logods <- log(prev/(1-prev)) ) # logodds(improvement)

# ROC MIC

rocobj <- roc(group ~ TScore_change, data = btsmp, quiet = TRUE)

cuty <- coords(rocobj, x="best", input="threshold", ret="threshold",

best.method="youden", transpose = TRUE)

( mic.roc.boot[i] <- cuty[sample(length(cuty),1)] ) # MIC(ROC-Youden)

# Predictive MIC

fit <- glm(group ~ TScore_change, data = btsmp, family = "binomial")

# summary(fit)

C <- coef(fit)[1] # intercept coefficient C

B <- coef(fit)[2] # regression coefficient B

( mic.pred.boot[i] <- mic.pred <- (logods-C)/B ) # MIC(pred)

Scf <- 0.09 * SD + 0.103 * SD * Cor

( mic.adj.boot[i] <- mic.pred - Scf * logods ) # MIC(adj)

}

### End of bootstrapping

# Results:

mean(mic.roc.boot) # MIC(ROC) mean of bootstraps

quantile(mic.roc.boot, c(0.05, 0.95)) # 90% CI

mean(mic.pred.boot) # MIC(pred) mean of bootstraps

quantile(mic.pred.boot, c(0.05, 0.95)) # 90% CI

mean(mic.adj.boot) # MIC(adj) mean of bootstraps

quantile(mic.adj.boot, c(0.05, 0.95)) # 90% CI

**Procedure to determine MIC_ROC_**

The aim of this analyses if to find the PROMIS change score that can optimally distinguish between patients who are improved and patients who are not improved, based on the anchor. A detailed example is provided by de Vet et al. [32].

Step 1. This step is the same for the MIC_predict_ and MIC_ROC_. Divide the study sample in two groups: improved versus not improved.

Step 2 (optional). It can be helpful to plot the distribution of change scores on the PROMIS measure of interest for the improved and not improved group, which provides insight in the ability of the anchor to discriminate between patients who are improved and patients who are not improved (example Figure 1). Instructions for creating the plot can be found [here](http://www.clinimetrics.nl/images/upload/files/Instructions%20anchor-based%20MIC%20distribution%202020.pdf). Use percentages instead of frequencies to avoid that the sample sizes of the two groups influence the curves and cut-off values.


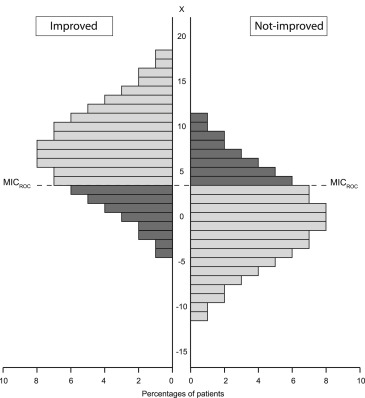


Figure 1. Distribution of a hypothetical sample of 100 improved and 100 not improved patients. The vertical axis depicts the PROMIS change score. MIC_ROC_ represents the MIC based on the optimal ROC cut-off point characterized by maximization of the sum of proportions correctly classified patients of both groups. The light shaded patients are correctly classified, whereas the dark shaded patients are misclassified [21].

Step 3. Perform a ROC analysis with the dichotomous group variable (improved versus not improved) as the ‘gold standard’ variable and the PROMIS change score as the ‘test’ variable (like in diagnostic testing). In a ROC analysis sensitivity and specificity for all possible change scores on the PROMIS measure are calculated and plotted.

Sensitivity refers to the proportion of improved patients (based on the anchor) what would be correctly identified as improved by the PROMIS measure when the associated change score would be used as MIC (percentage true positives). Specificity refers to the proportion of not improved patients that would be correctly identified as not improved by the PROMIS measure when the associated change score would be used as MIC (percentage true negatives). Next, calculate the proportion of false positives (1-sensitivity) and false-negatives (1-specificity) and add up these two proportions for all possible change scores on the PROMIS change score. Finally, to determine the MIC_ROC_ the PROMIS change score for which the value of ((1-sensitivity) + (1-sensitivity)) is lowest is taken (Example Table 1).

Calculating MIC_ROC_ with SPSS

In this syntax it is assumed that the group variable is coded as 0=not improved, 1=improved.

It is also assumed that larger change scores indicate improvement (TESTPOS=LARGE). If larger change scores indicate less improvement change LARGE to SMALL.

The analysis can be found in the menu under ”Analyze”, “Classify”, “ROC analysis”.

ROC ANALYSIS TScore_change BY group (1)

/MISSING USERMISSING=EXCLUDE

/CRITERIA CUTOFF=INCLUDE TESTPOS=LARGE DISTRIBUTION=FREE CI=95

/DESIGN PAIR=FALSE

/PLOT CURVE=ROC(REFERENCE) MODELQUALITY=FALSE

/PRINT SE=FALSE COORDINATES=ROC CLASSIFIER=FALSE.

The last part of step 3 should be done manually, e.g. by copying the SPSS Table to Excel.

Calculating MIC_ROC_ with R

See above.

**Online supplement 3**

This supplement belongs to the paper:

**Minimal Important Change (MIC): A conceptual clarification and systematic review of MIC estimates of PROMIS measures**

Caroline B Terwee, John Devin Peipert, Robert Chapman, Jin-Shei Lai, Berend Terluin, David Cella, Pip Griffith, Lidwine B Mokkink

**Pubmed search strategy**

We also included search terms to identify studies that estimated the minimal detectable change of PROMIS measures, for another paper (not published yet).

**PROMIS search terms**

PROMIS[tiab] OR “Patient-Reported Outcomes Measurement Information System”[tiab] OR “Patient-Reported Outcome Measurement Information System”[tiab] OR “Patient Reported Outcomes Measurement Information System”[tiab] OR “Patient Reported Outcome Measurement Information System”[tiab]

**AND**

**Test-retest reliability OR interpretability search terms**

"reproducibility of results"[MeSH] OR reproducib*[tiab] OR reliab*[tiab] OR unreliab*[tiab] OR agreement[tw] OR precision[tw] OR imprecision[tw] OR "precise values"[tw] OR test-retest[tiab] OR (test[tiab] AND retest[tiab]) OR (reliab*[tiab] AND (test[tiab] OR retest[tiab])) OR stability[tiab] OR interrater[tiab] OR inter-rater[tiab] OR intrarater[tiab] OR intra-rater[tiab] OR interobserver[tiab] OR inter-observer[tiab] OR intraobserver[tiab] OR intra-observer[tiab] OR interindividual[tiab] OR inter-individual[tiab] OR intraindividual[tiab] OR intra-individual[tiab] OR kappa[tiab] OR kappa's[tiab] OR kappas[tiab] OR repeatab*[tw] OR ((replicab*[tw] OR repeated[tw]) AND (measure[tw] OR measures[tw] OR findings[tw] OR result[tw] OR results[tw] OR test[tw] OR tests[tw])) OR generaliza*[tiab] OR generalisa*[tiab] OR (intraclass[tiab] AND correlation*[tiab]) OR error[tiab] OR errors[tiab] OR (uncertainty[tiab] AND (measurement[tiab] OR measuring[tiab])) OR "standard error of measurement"[tiab] OR (limit[tiab] AND detection[tiab]) OR interpretab*[tiab] OR ((minimal[tiab] OR minimally[tiab] OR clinical[tiab] OR clinically[tiab]) AND (important[tiab] OR significant[tiab] OR detectable[tiab]) AND (change[tiab] OR difference[tiab])) OR (small*[tiab] AND (real[tiab] OR detectable[tiab]) AND (change[tiab] OR difference[tiab])) OR "meaningful change"[tiab].
